# Informing Implementation: Perspectives from the Australian University Community Regarding an Animal Assisted Intervention

**DOI:** 10.3390/ani12243569

**Published:** 2022-12-16

**Authors:** Emily Cooke, Claire Henderson-Wilson, Elyse Warner, Anthony D. LaMontagne

**Affiliations:** School of Health and Social Development, Faculty of Health, Deakin University, Burwood, VIC 3125, Australia

**Keywords:** animal assisted intervention, university students, university staff members, implementation research

## Abstract

**Simple Summary:**

Animal Assisted Interventions have become increasingly popular in the university setting; however, there is limited research exploring their potential on an Australian university campus and participants’ views prior to implementation. Therefore, this study aimed to explore university staff members’ and students’ interest in participating in an Animal Assisted Intervention and their perspectives on intervention characteristics. Participants indicated their preferred intervention characteristics for the location of the intervention, frequency of participating, and ways of hearing about the intervention. Participants also discussed various considerations that may impact the intervention (such as the accessibility of the location, the impact of workload on participating, the effectiveness of promotion strategies, and factors that may assist therapy animal welfare, such as the therapy animals’ handlers). Gaining an insight into the university community’s views prior to implementation may ensure the intervention is feasible to implement and can be beneficial to both humans and therapy animals.

**Abstract:**

Animal Assisted Interventions (AAIs) have become increasingly popular in the university setting; however, there is limited research exploring their potential on an Australian university campus and participants’ views prior to implementation. Therefore, this study aimed to explore university staff members’ and students’ interest in participating in an AAI and their perspectives on intervention characteristics. This was a mixed methods study, using an online survey and semi-structured interviews. The survey had 344 responses, and 45 interviews were conducted. A large majority of participants (86%) were interested in participating in an AAI. In the survey, participants indicated their preferred intervention characteristics for the location of the intervention, frequency of participating, and ways of hearing about the intervention. Participants also expressed concerns regarding therapy animal welfare. In interviews, participants discussed various considerations which may impact the intervention (such as the accessibility of the location, the impact of workload on participating, the effectiveness of promotion strategies, and factors that may assist therapy animal welfare, such as the therapy animals’ handlers). Gaining an insight into the university community’s views prior to implementation may ensure the intervention is feasible to implement and can be beneficial to both humans and therapy animals.

## 1. Introduction

Animal Assisted Interventions (AAIs) involve trained therapy animals and their handlers entering a setting to provide support to the people within it [1]. In the past decade, AAIs have gained popularity in universities in the United States, Canada, the United Kingdom, and throughout Europe [2]. Several studies have assessed the impact of an AAI on students’ levels of stress, anxiety, mood, and campus connectedness and found that an AAI can positively influence these outcomes [2]. Given the rising levels of stress and anxiety experienced by university students globally [3,4], an AAI could provide one means to reduce stress levels in university settings.

Compared to the number of studies exploring the outcomes of AAIs for university students, there is little research exploring how these interventions are implemented to promote future enactment. Some informal studies recount their processes for implementation in a university counselling centre or library [1,5,6,7,8,9,10,11]. A recent book on AAIs in the university setting has also included some details of implementation [12]. However, more research into how AAIs are developed is required to enhance future implementation.

There are currently no studies that explore potential AAI participants’ views regarding the implementation of a program on campus. There is also a limited exploration into potential participants’ interest in participating in an AAI on campus or the desired frequency of participation. Likewise, there is limited research exploring an AAI on an Australian university campus. This is significant, given the rates of increased stress and anxiety experienced by Australian university students [13,14] and staff members [15,16]. Therefore, this research aimed to explore Australian university staff members’ and students’ perspectives towards an AAI on campus in order to inform implementation. This aim was guided by two research questions:

1. How interested are students and staff members in participating in an AAI on campus?
2. What are students’ and staff members’ perspectives regarding the intervention characteristics of an AAI on campus?

## 2. Materials and Methods

### 2.1. Study Design

This research was an explanatory mixed methods study [17]. This study design was selected to first gain an overview of perspectives from the university community and subsequently explore some perspectives in greater depth in order to provide an explanation for responses. Survey data were utilised to gain an overview of students’ and staff members’ perspectives, and interview data examined these perspectives in more detail [17].

### 2.2. Theoretical Framework

**Figure 1:**
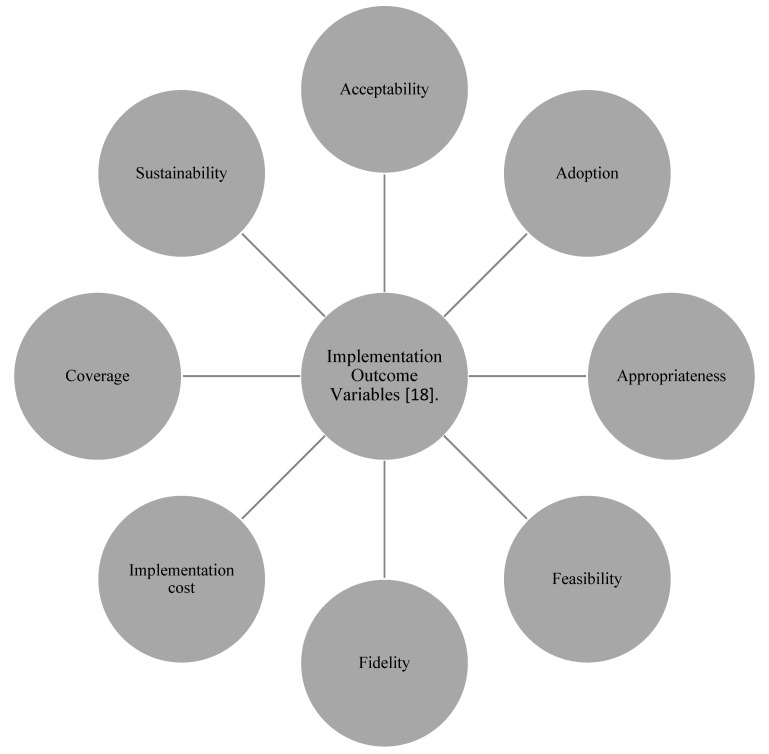
Implementation Outcome Variables [18].

This study was theoretically underpinned by the “Implementation Outcome Variables” framework, which can be utilised to assess the effectiveness of program implementation [18]. Therefore, comparing how participants’ views align with these variables can help to shape successful AAI implementation. Figure 1 illustrates the Implementation Outcome Variables.

### 2.3. Sampling and Recruitment

Both student and staff member surveys and interviews received ethics approval from the university’s Human Ethics Advisory Group. Stratified random sampling was conducted with the aim of obtaining a representative sample across the university’s faculties [19].

Student survey:

Twelve courses (3 undergraduate or postgraduate courses from each faculty) from the selected Australian university were selected at random through a random number generator. A first-year, second-year, and third-year unit from each course was then selected at random using a random number generator (36 subjects in total). The Heads of these subjects were asked via email if they would post the survey flyer and link on their subject site. If they declined, a different unit was randomly selected. Recruitment also occurred through the Student Union Association’s social media accounts.

Staff member survey:

Faculty or division heads at the selected university were asked via email whether they would share the survey with their staff members. Inclusion criteria included those aged 18 years or older who spoke English and were currently employed by the university or enrolled in a course at the university.

Follow-up interviews:

The survey included a question regarding interest in participating in a qualitative interview. If participants were interested, they were redirected to another Qualtrics survey asking for their email address to receive a recruitment email regarding interview participation. The Plain Language Statement and Consent Form was attached to this email to ensure informed consent was obtained.

### 2.4. Data Collection & Measures

The first phase of the research involved conducting an anonymous survey with students and staff members from the university. Within the survey, participants were provided with a description of an AAI, which stated, “for this research, AAIs involve bringing trained support animals and their handlers into a particular setting where they can provide support to the individuals within it [1]” in order to ensure they understood the intervention was regarding trained therapy animals and did not misinterpret it to be regarding companion animals. Survey questions related to this study included interest in an AAI on campus, how often participants would be interested in participating, and details regarding preferred intervention characteristics. Examples of survey questions include: “Where would you like to participate in an AAI on campus? Please select all that apply” and “How often would you participate in an AAI on campus?” A pilot study was conducted prior to survey circulation with six master’s and/or PhD students to ensure the survey was consistent and engaging. The student survey was distributed in August–September 2021, and the staff member survey was distributed in September–October 2021. Twenty-five Subject Heads shared the survey link on their subject sites. Seventeen faculties and administrative divisions (*n* = 4 faculties, *n* = 13 divisions) sent the survey to their staff (either by direct email or incorporated within a newsletter). Forty-seven (*n* = 24 staff members, *n* = 23 students) responses were omitted from the analysis due to incompleteness (participants were told if they wished to withdraw their survey response to close the browser prior to submitting the final response). There was a total of 344 survey responses (194 students and 150 staff members). This was a response rate of around 4% for students and 3% for staff members. As a token of appreciation, students were given the option to enter a chance to win one of thirty $20 food vouchers if they completed the survey. This was not offered to staff members as it is against the university’s policy for staff members to accept gifts from students (data collection was completed by the student researcher).

The second phase of research was semi-structured interviews. Interviews were conducted online over Zoom, ranged from 20–75 min (average length was 40 min), and were recorded with the participants’ consent. One hundred and ten people (*n* = 70 students, *n* = 40 staff members) were contacted regarding their expressed interest in participating in an interview. A total of 45 participants (*n* = 24 students, *n* = 21 staff members) completed qualitative interviews. This was a response rate of 41% (34% for students and 50% for staff). Interview questions included expanding on survey responses and whether participants had any further perspectives regarding intervention characteristics. Some examples of questions from the interview guide include: “You mentioned that you would participate in an AAI on campus [selected frequency]; could you please tell me why you think this would be the most beneficial for you?” and “Could you please tell me why [selected promotion responses] would be the best way for you to find out about the intervention?” Student participants received a $20 food voucher as a token of appreciation. Participants were given the opportunity to review their transcripts prior to analysis, with seven participants (*n* = 1 student, *n* = 6 staff members) editing for clarity.

### 2.5. Data Analysis

Survey data descriptive frequencies were calculated in SPSS. Thematic analysis was utilised for interview data [20]. The first author transcribed interviews and imported them into NVivo (v20 1.6.1). Data immersion then occurred by reading transcripts multiple times. Initial coding was recorded in NVivo, with the second and third authors reviewing codes and suggesting further refinement. These codes were consolidated to generate subthemes and themes, which were cross-checked against research questions and survey data to ensure they addressed the study’s aim. Staff member interview data were coded first. Codes were adjusted once student interview data had also been coded to ensure themes and subthemes reflected both cohorts’ data, as there were minimal differences between the two cohorts. Due to qualitative data being utilised to explain survey data in more detail, both inductive and deductive coding was used.

## 3. Results

Table 1 displays the demographic data of participants.

### 3.1. Interest in Participating

Eighty-six per cent (*n* = 299; *n* = 176 students, *n* = 123 staff members) of survey participants were interested in an AAI. Of the 10% of the sample who had participated in an AAI before (*n* = 36; *n* = 17 students, *n* = 19 staff members), 92% (*n* = 33; *n* = 16 students, *n* = 17 staff members) were interested in participating again.

#### Structure of the AAI 

Several staff members and students suggested individual AAI sessions maybe be more beneficial for those with higher needs, but group sessions may serve well to reduce stress. One staff member and some students expressed interest in individual sessions, either due to being shy or the desire to have a break from social interaction.

*“I think I’d probably prefer individual just because … I feel like I’d probably be a little bit more shy if it was in a group, especially if it’s a group of people I don’t know”*

(P15-student)

Other staff members expressed interest in either individual or group sessions. Some students highlighted that group sessions would have the biggest impact, given that they could target more of the university community at once. It was also mentioned that the cost of individual sessions might outweigh the benefits.

The thematic analysis led to the development of four themes (accessible location, available time, awareness of intervention, and therapy animal wellbeing). Each theme relates to survey data.

### 3.2. Accessible Location

This theme relates to preferred location survey data. Table 2 displays data on participants’ preferred location for an AAI and indicates the most selected option was ‘outside’ for students and ‘student central’ (an undercover area for socialising, eating, and student administration queries) for staff members (outside was the most selected overall (52%, *n* = 180)). Twelve of the 27 (44%) ‘other’ responses were related to specific office buildings or a roaming intervention.

#### 3.2.1. Ability to Be Contained

Interview participants raised the idea that the intervention’s ability to be contained would be an important consideration. Some participants suggested the intervention should not be contained within a building due to COVID-19 concerns. Contrastingly, other participants mentioned that hosting the intervention in a building might be more beneficial, as organisers could control the number of people entering, which might make the intervention less overwhelming for both human participants and therapy animals.

*I think inside is able to be controlled a bit more, and you can control the environment around it with more people coming in and then like going through the space or if it’s just like able to be quiet*

(P16-student)

#### 3.2.2. Accessibility

Participants highlighted that an intervention in an easily accessible location might lead to increased participation. One staff member suggested an intervention that came into certain office buildings would be more accessible for staff. Other staff members agreed with this or stated that this was their assumption regarding the intervention.

Both cohorts acknowledged that the easiest location to find on campus would be student central. Students mentioned that a lot of people pass through the area, and it is not a study space, which could increase participation.

*“Just a good hub central point … because in my experience I’m not familiar with all the other buildings on campus, so for me those buildings where everybody would know where they are”*

(P21-staff member)

Contrastingly, two staff members highlighted the idea that a very public space, such as student central, might make it difficult for those with allergies or phobias to avoid. It was acknowledged that the intervention would need to be accessible for those interested in participating and avoidable for those not interested.

#### 3.2.3. Visibility

Some participants stated that a highly visible location, such as outside or student central, might lead to increased participation, as these spots have high traffic.

*[Student central is] the watering hole of the uni in a sense … like pretty much everyone would eventually pass by … anyway, … even if you don’t get a huge turnout of people who were initially wanting to come, you’ll definitely have people in the general area who will be on looking and seeing what’s happening, and they might be keen to join*

(P7-student)

However, this could be a drawback as it could lead to too many people approaching the intervention, making it overwhelming for the participants and therapy animals involved.

*If it was outside, obviously, people would be able to see it and want to get involved, but that could also be a negative because you might have people just joining up, so I used to run some educational tours where I had a dog with me, and people would see us and just try to come up and pet the dog when we already had a specific group of people who were there to do that, and that could then overwhelm the dog*

(P14-staff member)

### 3.3. Available Time

This theme relates to the preferred frequency of an AAI. Table 3 displays data on this, indicating that the most selected option was weekly for both students and staff members (34%, *n* = 119).

Staff members indicated that workload was the largest determining factor in selecting a frequency for participation. It was acknowledged that if the intervention were too frequent, it might apply extra pressure on those interested in participating, so fortnightly or monthly might be more suitable.

*I think anything more frequent than [monthly] would apply pressure in terms of time and availability … I think it’d be great to do something every fortnight, but I think with these things… I liken it to perhaps a wellbeing initiative, where you might have a meditation session or a yoga session on site, and people love the idea, and they’ll sign up for it, but in that moment to actually stop working and …go participate, it doesn’t always get the buy-in that people commit to*

(P12-staff member)

Contrastingly, other staff members mentioned that they would make time in their schedule to attend a weekly intervention. This was due to their having a natural affinity for animals or ease of scheduling a regular time.

Similarly, a majority of students mentioned, in interviews, that they would prefer the intervention within the second half of the semester when their workloads and stress levels were higher. Around exam time or the end of the semester were the most popular suggestions.

*Maybe week 10 or maybe earlier at 9 because that’s the time you probably have your final assignments or somewhere closer to that exams coming up, and you probably need a dose of relaxation before that, but alternatively… it would be beneficial to have … during those exam hours … [students] could come after their exams to de-stress because exam gets you kind of worked up and anxious*

(P1-student)

Contrastingly, some staff members and students raised the fact that when they are feeling quite stressed or overwhelmed (which is when they acknowledged they might benefit the most from an AAI), they may be less likely to participate due to these feelings. Participants recognised that this might impact participation rates, as people on campus might be unable to attend the intervention when they could potentially benefit the most from it.

Several students mentioned having the intervention around lunchtime, when students have a break from classes or studying and, therefore, would be available to participate. However, as mentioned by one particular student, to capture a large amount of the cohort, the intervention might need to run for several hours so students could work it into their timetables. Another student highlighted the complexity of catering to the student cohort by discussing the fact that there are part-time students who work full-time hours and study off campus who may also be interested in participating. Finally, one other student highlighted the notion that the cost of the intervention would be a factor in determining its frequency on campus.

### 3.4. Awareness of Intervention

This theme relates to participants’ preferred way of finding out about the intervention. Table 4 indicates that the most selected option was email for both students and staff members (80.8%, *n* = 278).

#### 3.4.1. Efficient Strategies for Promotion

Most students and staff members mentioned, in interviews, that email would be the easiest way to find out about an AAI, as they were always checking their emails. It was acknowledged that this would be the easiest way to inform everyone on campus when the intervention was occurring.

Students and staff members also suggested that social media would be an effective way to promote the intervention, as they checked it frequently. However, another staff member noted that they did not follow the university on social media as they tried to keep their work life separate.

Several participants suggested that seeing the therapy dogs on campus would help promote the intervention. As noted by one staff member:

*“Seeing them there would be another great way to sort of be aware of what’s going on … some people just can’t help themselves; they see a dog, they want to go and say hi”*

(P10-staff member)

Many participants mentioned that they did not notice flyers on campus. However, other participants stated that they either took the time to look at flyers on campus or noticed flyers if they were bright and stood out. It was noted that flyers near the interventions’ location would be beneficial. A few participants suggested that a combination of multiple promotion strategies might be the best way to ensure the university population found out about the intervention, either so they could attend if they were interested or avoid the area if they were not.

#### 3.4.2. What to Include in Promotion Material

Some participants suggested information that could be included in promotional material. The most suggested point was ensuring that the material indicated therapy dogs and their handlers were trained, so people did not misinterpret the intervention as being one at which they would be able to bring their own companion animal.

*That’s the thing that worries me a little bit when they talk about these programs is that they’re not highlighting or differentiating those … it’s about bringing pets on campus or in the workplace versus a very sort of structured way of doing it with a level of trust involved and responsibility*

(P6-staff member)

Another point was ensuring that details regarding where and when the intervention would take place would need to be disseminated well ahead of time so that those with allergies or phobias could avoid the area. One staff member suggested mentioning that appropriate cleaning would take place afterwards to further put those with allergies at ease. One student highlighted the idea that including details about the therapy dog’s health and the availability of hand sanitiser might help those who felt uneasy being in a group because of COVID-19.

### 3.5. Therapy Animal Wellbeing

This theme relates to the survey participants’ largest concern (besides not having any concerns), namely, that the therapy animals involved might end up tired or stressed (n = 120; *n* = 76 students, *n* = 44 staff members).

#### 3.5.1. Assumed Welfare in AAI

Several interview participants highlighted that they placed the responsibility for the therapy animal’s welfare on their handler, as they assumed handlers were trained advocates for their therapy animals and were aware of the signs of fatigue or stress in their therapy animals.

Some staff members assumed that because the vast majority of people on campus were adults, they might interact with the therapy animals more calmly than a group of children would, which would aid in therapy animal welfare. One student assumed that if the intervention were on campus, it would have had to go through a lengthy approval process, reassuring them that the intervention has considered animal welfare.

*I’m sure they are coming from a good from an organisation … and [the university] would have to approve it probably through ethics committee or something like that so you can make an educated guess it’s probably all above board*

(P11-student)

#### 3.5.2. Therapy Animal Personality

Participants highlighted that the personality of the therapy animals could aid their welfare. For example, one participant suggested that animals that were highly social would be better suited to the role of a therapy animal. Other participants mentioned that the animals involved would be those that enjoyed attention from unfamiliar people. Participants also suggested that therapy dogs might be the best option, as cats might be less sociable and therefore become stressed in an AAI more easily.

*“I feel like dogs are the main thing, whereas if you’ve got like cats and rabbits and stuff, they could be quite fearful of what’s going on”*

(P20-staff member)

#### 3.5.3. Intervention Needs to Be Beneficial for the Therapy Animals Involved

Some participants highlighted the idea that the intervention would need to be beneficial for the therapy animals involved. It was acknowledged that the potential stress-lowering benefits of the intervention were redundant if they were causing stress to the therapy animals. One staff member mentioned that they were not a fan of AAIs, as they were predominately for human benefit. They noted that the therapy animals needed to be considered in the development and not utilised as an object/tool.

*I think that we need to seek consent, and I think that dogs, there would be … plenty of dogs that are probably good and would love to … come to campus, they get to meet lots of people, but there’s also lots of dogs who would not deal with that*

(P11-staff member)

Several considerations for therapy animal welfare were raised by students and staff members. Some participants noted that there would need to be fresh water and a space nearby for therapy animals to have a break. Considerations would also need to be made regarding the number of people interacting with the therapy dog and how to mitigate that so that the therapy dog does not feel overwhelmed. Participants suggested the number of hours therapy animals worked would need to be considered, and the frequency of the intervention on campus could work in the therapy animals’ favour (i.e., fortnightly may be better for their welfare than weekly).

Several staff members and students mentioned that those participating in the intervention might need training on how to interact with therapy animals, and handlers could provide this. This training could increase the enjoyment therapy animals get out of the intervention. The handler could also teach participants the visual cues of fatigue or stress in the therapy animals.

*I probably haven’t spent a lot of time around dogs as much as an adult, and I think, for me, that would be a good way of actually having some interaction that’s going to be a positive one and maybe even learning a little bit about how to interact with that animal in a way that, is right for the animal so, that …if we did ever get a dog or something in my personal life, I think that that could be a benefit as well*

(P18-staff member)

The findings from this study can be applied to all of the Implementation Outcome Variables [18]. Table 5 displays how this study’s findings align with these variables.

## 4. Discussion

This study aimed to explore staff and student interest in, and perspectives of, characteristics of an AAI on campus. Overall, a large majority of participants were interested in participating in an AAI. This mirrors previous studies, with one indicating that 96% of first-year students would be interested in an AAI on campus [21]. Other studies have illustrated that AAIs on campus were quite popular interventions [5,7,8,10]. Several studies have highlighted how, due to the popularity of the intervention on campus, programs have been able to expand or be implemented more frequently, suggesting that the popularity could play a role in the intervention’s sustainability [5,7,8]. In regard to locations, previous studies have hosted their interventions in libraries [1,9,10], counselling centres [5,6,7], student centres [22], classrooms [23], and residence halls [24], and another program roamed and visited students at several locations [25]. Reasons for these locations included promoting awareness of counselling centres [5,7,8], ensuring the location was large enough, and weather considerations [5]. The question of whether ‘outside’ could be a successful location has yet to be explored in detail, which may be an important consideration, especially with campus reactivations post-COVID-19 lockdowns, as raised by some participants.

Participants’ views regarding the preferred time to run an AAI for students reflect results from previous studies that conducted their AAIs around exam time, as this was noted as the most stressful period for students [9,25,26,27,28,29]. Other researchers held their AAIs weekly, reflecting this study’s findings that a weekly intervention would be preferred [30,31]. One study started its AAI at lunchtime [5], reflecting students’ views in the current study, which suggested that lunchtime might be a suitable time to ensure increased participation.

Regarding promoting the intervention, previous studies have promoted the intervention through social media [5,7], university calendars [5], flyers [5,7], the library website [9], and televisions throughout the university [7,9]. This reflects the views of participants in the current study, namely that promotion through multiple channels could be the most effective way to ensure the university community was aware of the intervention. Echoing some participants’ views regarding the effectiveness of flyers, another study used flyers and social media and found the flyers were not as effective as social media and word of mouth [1]. Another study promoted AAIs via email but found that most of their participants attended the intervention because they saw it on campus [27]. This supports participants’ views regarding seeing the dogs on campus and how that could be an additional strategy to encourage participants to find out about the intervention and that a highly accessible or visible location might lead to increased participation. However, as mentioned by some participants in this study, doing so may also lead to increased stress levels for the therapy dogs [32].

Participants’ concerns regarding therapy animal welfare reflect the current literature. Several recent studies have considered the wellbeing of therapy dogs involved in AAIs [33,34,35,36,37]. One study suggested handlers play a large role in therapy animal wellbeing and highlighted the fact that one organisation uses the phrase “YAYABA” (You Are Your Animal’s Best Advocate). [33] (p. 5). Another recent book has noted that the main role of handlers is to advocate for their therapy animal and to ensure they are removed from any scenarios where they begin to show signs of stress, reflecting this study’s participants’ assumptions regarding the handler’s role in an AAI [32].

Participants’ perspectives on dogs being the most suitable therapy animal are reflected in the literature, with dogs being the most common therapy animal entering university campuses [2]. One study highlighted that therapy dogs were assessed on how comfortable they were around people, other dogs, and noisy environments as a part of their training to become therapy dogs [7]. Another study measured therapy dogs’ cortisol before and after an AAI in a mental healthcare facility and found that, overall, the therapy dogs were not stressed by the AAI [35]. Additionally, a study in the university setting had handlers and a separate animal behaviourist examine therapy dogs’ behaviour and found that a quarter had increased stress, a quarter had decreased stress, and the remaining half showed no change in stress levels following the AAI [31]. These authors also found that the biggest factor influencing therapy dog stress was their handlers’ stress levels prior to the intervention, suggesting that handlers play a big role in the therapy animals’ welfare [31]. A recent study suggests that therapy animals should be able to “opt in and opt out,” similarly to human participants [36] (p. 4). Likewise, a recent book suggests including a consent test, which involves the human petting the therapy dog, stopping this interaction, and seeing if the therapy dog attempts to make the interaction begin again [32]. However, further research is required to determine a standardised way of ensuring therapy dogs are enjoying the AAI as much as human participants.

### Strengths and Limitations

The strengths of this research are that it includes both staff members’ and students’ perspectives regarding interest in and details of characteristics of an AAI on campus. This study also offers an Australian university perspective, which has limited exploration in the existing research. Furthermore, this study considers details regarding implementing an AAI on campus, which has not been discussed in the literature in much detail [1,5,6,7,8,9,10,11,12] compared to the number of studies that discuss the outcomes of an AAI on campus [2]. This is important, as successful outcomes associated with an AAI can only occur if the intervention is implemented successfully. This study is the first to apply details of an AAI to the Implementation Outcome Variables [18]. This is significant, as these variables are fundamental elements to consider when implementing an intervention and aid in shaping the intervention’s success [18]. By relating the findings of this study to these variables, the study can provide guidance on factors and potential strategies to consider prior to implementing an AAI on campus, which may enhance the success of implementation. This research is not without limitations. First, despite promotion on subject sites, the student associations’ social media pages, in newsletters, and some faculty/division heads emailing the survey directly to their staff, this study’s survey had a low response rate. This may have been due in part to ethical and COVID-19 lockdown restrictions precluding the use of additional strategies, such as flyers on campus, presentations in lectures/seminars, additional social media or Microsoft Teams promotion, or directly emailing students and staff members the survey and sending follow up reminders to complete it. As the survey was on an AAI, individuals that are animal-orientated were more likely to respond, resulting in a selection bias. However, companion animal ownership rates in this study were similar to that of the Australian population (67% of participants in this study currently had a companion animal, 95% had a companion animal before, 60% of the Australian population had a companion animal in 2019, 90% had a companion animal before) [38]. However, perspectives from those with less positive views of dogs were also gained. This study also may not be generalisable to other universities.

## 5. Conclusions and Implications

The majority of participants in this study were interested in participating in an AAI. Participants also raised several ideas and suggestions regarding intervention characteristics. In regard to the location, participants suggested that its ability to be contained, along with how accessible and visible it was on campus, were important factors to consider. Participants also highlighted that the largest factor contributing to how often they would engage with an AAI on campus was their workload, which they also acknowledged would be when they might benefit the most from the intervention. Several methods for promoting the AAI were discussed by participants, with the majority recommending a combination of email and social media as efficient strategies. It was also emphasised that the AAI would need to be beneficial for the therapy animals as well as human participants. These findings highlight important considerations related to implementing a successful AAI on campus.

To further determine the most successful way to implement a sustainable AAI, more studies exploring the impact of intervention characteristics on program attendance and enjoyment, and more studies asking participants how they found out about the intervention, are needed. More studies should publish protocol papers detailing implementation with lessons learnt to enhance the success of future interventions. Additionally, future research should use the Implementation Outcome Variables to examine the success of AAI implementation. Further research should also be conducted into assessing whether AAIs are beneficial for the therapy animals involved, and more research regarding therapy animals consenting to participate in an AAI. A summary of these recommendations is presented in Table 6. Further research will determine the most successful way to sustainably implement an AAI on an Australian university campus to enhance student and staff member wellbeing.

## Figures and Tables

**Table 1:** Demographic data of participants.

Age Range	Survey	Interviews
18–25	154 (*n* = 143 students, *n* = 11 staff members)	15 (*n* = 15 students)
26–35	70 (*n* = 32 students, *n* = 38 staff members)	17 (*n* = 7 students, *n* = 10 staff members)
36–45	59 (*n* = 13 students, *n* = 46 staff members)	6 (*n* = 1 student, *n* = 5 staff members)
46–55	44 (*n* = 4 students, *n* = 40 staff members)	5 (*n* = 5 staff members)
56–65	14 (*n* = 2 students, *n* = 12 staff members)	2 (*n* = 1 student, *n* = 1 staff member)
66–75	2 (*n* = 2 staff members)	-
No response	1 (*n* = 1 staff member)	-
**Gender**		
Female	265 (*n* = 156 students, *n* = 109 staff members)	32 (*n* = 16 students, *n* = 16 staff members)
Male	73 (*n* = 34 students, *n* = 39 staff members)	12 (*n* = 7 students, *n* = 5 staff members)
Third gender/non-binary	3 (*n* = 3 students)	1 (*n* = 1 student)
Prefer not to say	3 (*n* = 1 student, *n* = 2 staff members)	-
**Faculty/Administrative Division**		
Arts and Education	53 (*n* = 51 students, *n* = 2 staff members)	7 (*n* = 7 students)
Business and Law	22 (*n* = 21 students, *n* = 1 staff member)	4 (*n* = 3 students, *n* = 1 staff members)
Health	68 (*n*= 57 students, *n* = 11 staff members)	9 (*n* = 7 students, *n* = 2 staff members)
Science	109 (*n* = 65 students, *n* = 44 staff members)	14 (*n* = 7 students, *n* = 7 staff members)
Stakeholder relationships	6 (*n* = 6 staff members)	2 (*n* = 2 staff members)
Student Administration	40 (*n* = 40 staff members)	5 (*n* = 5 staff members)
Equity and Inclusion	9 (*n* = 9 staff members)	2 (*n* = 2 staff members)
Research	7 (*n* = 7 staff members)	1 (*n* = 1 staff member)
Student Residential Services	7 (*n* = 7 staff members)	1 (*n* = 1 staff member)
Vice Chancellor’s Office	1 (*n* = 1 staff member)	-
General Counsel	2 (*n* = 2 staff members)	-
Marketing	8 (*n* = 8 staff members)	-
Record preservation	1 (*n* = 1 staff member)	-
Facilities Maintenance	3 (*n* = 3 staff members)	-
Teaching Support Services	3 (*n* = 3 staff members)	-
No response	5 (*n* = 5 staff members)	
**Currently has a companion animal**		
Yes	232 (*n* = 121 students, *n* = 111 staff members)	27 (*n* = 14 students, *n* = 13 staff members)
No	111 (*n* = 72 students, *n* = 39 staff members)	17 (*n* = 9 students, *n* = 8 staff members)
No response	1 (*n* = 1 student)	1 (*n* = 1 student)
**Has had a companion animal before (from those that did not currently)**		
Yes	98 (*n* = 61 students, *n* = 37 staff members)	14 (*n* = 6 students, *n* = 8 staff members)
No	13 (*n* = 11 students, *n* = 2 staff members)	3 (*n* = 3 students)
No response	1 (*n* = 1 student)	1 (*n* = 1 student)

**Table 2:** location survey data.

Where Would You Like to Participate in an Animal Assisted Intervention (AAI) on Campus? Please Select All That Apply.	Students	Staff Members	Total
Library	*n* = 97 (50%)	*n* = 52 (34.6%)	*n* = 149 (43.3%)
Student Central	*n* = 95 (48.9%)	*n* = 81 (54%)	*n* = 176 (50.2%)
Student Union Association	*n* = 87 (44.8%)	*n* = 39 (26%)	*n* = 126 (36.6%)
Health and Wellbeing Centre	*n* = 99 (51%)	*n* = 63 (42%)	*n* = 162 (47%)
Student Residences	*n* = 60 (30.9%)	*n* = 35 (23.3%)	*n* = 95 (27.6%)
Outside near one of these locations	*n* = 108 (55.6%)	*n* = 72 (48%)	*n* = 180 (52.3%)
A less central area	*n* = 35 (18%)	*n* = 16 (10.6%)	*n* = 51 (14.8%)
Other	*n* = 7 (3.6%)	*n* = 20 (13.3%)	*n* = 27 (7.8%)

**Table 3:** preferred frequency survey data.

How Often Would You Participate in an AAI on Campus?	Students	Staff Members	Total
Fortnightly	*n* = 36 (18.5%)	*n* = 24 (16%)	*n* = 60 (17.4%)
Monthly	*n* = 29 (14.9%)	*n* = 25 (16.6%)	*n* = 54 (15.6%)
Never	*n* = 22 (11.3%)	*n* = 28 (18.6%)	*n* = 50 (14.5%)
Once or twice a semester	*n* = 35 (18%)	*n* = 26 (17.3%)	*n* = 61 (17.7%)
Weekly	*n* = 72 (37.1%)	*n* = 47 (31.3%)	*n* = 119 (34.5%)

**Table 4:** Preferred way to find out about an AAI on campus.

How Would You Like to Find Out About the AAI on Campus? Please Select All That Apply.	Students	Staff Members	Total
Email	*n* = 156 (80.4%)	*n* = 122 (81.3%)	*n* = 278 (80.8%)
Flyers on campus	*n* = 69 (35.5%)	*n* = 38 (25.3%)	*n* = 107 (49.4%)
Social media	*n* = 131 (67.5%)	*n* = 53 (35.3%)	*n* = 184 (53.4%)
By seeing the dogs on campus	*n* = 99 (51%)	*n* = 80 (53.3%)	*n* = 179 (52%)
Other	*n* = 1 (5.1%)	*n* = 6 (4%)	*n* = 7 (2%)

**Table 5:** study’s alignment with the Implementation Outcome Variables [18].

Implementation Outcome Variable [18]	Definition [18] (p. 5)	This Study’s Findings
Acceptability	“The perception among stakeholders (for example, consumers, providers, managers, policymakers) that an intervention is agreeable.”	Provides an overview of opinions from potential future consumers and determines their interest in an AAI on campus.
Adoption	“The intention, initial decision, or action to try to employ a new intervention.”	Highlights whether there is interest among the university community to try to employ an AAI on campus.
Appropriateness	“The perceived fit or relevance of the intervention in a particular setting or for a particular target audience (for example, provider or consumer) or problem.”	Illustrates whether those within the university setting are interested in participating in an AAI on campus and explores their perspectives on where on campus this could be the most suitable.
Feasibility	“The extent to which an intervention can be carried out in a particular setting or organisation.”	Provides perspectives from the university community regarding locations on campus where an AAI may be feasible. It also provides insight into how often those in the university community may attend and potential considerations regarding therapy animal welfare, which may further increase feasibility.
Fidelity	“The degree to which an intervention was implemented as it was designed in an original protocol, plan, or policy.”	Meticulous planning and careful consideration regarding details of intervention characteristics and therapy animal welfare (as raised by participants in this study) could aid in ensuring the AAI on campus is implemented according to plan.
Implementation cost	“The incremental cost of the implementation strategy (for example, how the services are delivered in a particular setting). The total cost of implementation would also include the cost of the intervention itself.”	One participant discussed how the cost would determine frequency of the intervention on campus. It was also mentioned that the cost of hosting individual sessions might outweigh the potential benefits, given that group sessions could target more of the university community at once.
Coverage	“The degree to which the population that is eligible to benefit from an intervention actually receives it.”	Participants in this study provided their perspectives on promotion strategies that could be effective, along with hesitations towards other promotion strategies, in order to increase the coverage. It was also mentioned that the location could lead to increased coverage.
Sustainability	“The extent to which an intervention is maintained or institutionalised in a given setting.”	Participants discussed various considerations which could lead to a more sustainable intervention (the location, considering those that are not interested, the frequency, promotion and the welfare of the therapy animals involved).

**Table 6:** summary of recommendations.

Further Research is Required:
Examining the impact of intervention characteristics on program attendance and enjoyment
Examining the effectiveness of various promotion strategies
Publishing details of implementation with lessons learnt in protocol papers
Using the Implementation Outcome Variables to examine the success of AAI implementation.
Assessing whether AAIs are beneficial for the therapy animals involved
Investigating therapy animal consent to participation within an AAI

## Data Availability

Data is not available due to ethical restrictions. Participants of this study did not consent for their data to be shared publicly.
